# A comprehensive nomogram for assessing the prognosis of non-small cell lung cancer patients receiving immunotherapy: a prospective cohort study in China

**DOI:** 10.3389/fimmu.2024.1487078

**Published:** 2024-11-20

**Authors:** Hongmei Li, Yuliang Yuan, Qianjie Xu, Guangzhong Liang, Zuhai Hu, Xiaosheng Li, Wei Zhang, Haike Lei

**Affiliations:** ^1^ Chongqing Key Laboratory of Translational Research for Cancer Metastasis and Individualized Treatment, Chongqing University Cancer Hospital, Chongqing, China; ^2^ Chongqing Cancer Multi-omics Big Data Application Engineering Research Center, Chongqing University Cancer Hospital, Chongqing, China; ^3^ Department of Health Statistics, School of Public Health, Chongqing Medical University, Chongqing, China

**Keywords:** NSCLC, PD-1/PD-L1, ICIS, nomogram, prognosis

## Abstract

**Objective:**

In China, lung cancer ranks first in both incidence and mortality among all malignant tumors. Non-small cell lung cancer (NSCLC) constitutes the vast majority of cases, accounting for 80% to 85% of cases. Immune checkpoint inhibitors (ICIs), either as monotherapies or combined with other treatments, have become the standard first-line therapy for NSCLC patients. This study aimed to establish a nomogram model for NSCLC patients receiving immunotherapy incorporating demographic information, clinical characteristics, and laboratory indicators.

**Methods:**

From January 1, 2019, to December 31, 2022, a prospective longitudinal cohort study involving 1321 patients with NSCLC undergoing immunotherapy was conducted at Chongqing University Cancer Hospital. Clinical and pathological characteristics, as well as follow-up data, were collected and analyzed. To explore prognostic factors affecting overall survival (OS), a Cox regression model was used to test the significance of various variables. Independent prognostic indicators were identified through multivariate analysis and then used to construct a nomogram prediction model. To validate the accuracy and practicality of this model, the concordance index (C-index), area under the receiver operating characteristic curve (AUC), calibration curve, and decision curve analysis (DCA) were used to assess the predictive performance of the nomogram.

**Result:**

In the final model, 11 variables from the training cohort were identified as independent risk factors for patients with NSCLC: age, KPS score, BMI, diabetes, targeted therapy, Hb, WBC, LDH, CRP, PLR, and LMR. The C-index for OS in the training cohort was 0.717 (95% CI, 0.689–0.745) and 0.704 (95% CI, 0.660–0.750) in the validation cohort. Calibration curves for survival probability showed good concordance between the nomogram predictions and actual observations. The AUCs for 1-year, 2-year, and 3-year OS in the training cohort were 0.724, 0.764, and 0.79, respectively, and 0.725, 0.736, and 0.818 in the validation cohort. DCA demonstrated that the nomogram model had a greater overall net benefit.

**Conclusion:**

A prognostic model for OS in NSCLC patients receiving immunotherapy was established, providing a simple and reliable tool for predicting patient survival (https://icisnsclc.shinyapps.io/DynNomapp/). This model offers valuable guidance for clinicians in making treatment decisions and recommendations.

## Introduction

The global cancer statistics for 2022 reveal that lung cancer (1), following other cancers, has become the second most common cancer worldwide and is also the malignancy with the highest mortality rate. Lung cancer accounts for 21% of all cancer-related deaths (2), making it the leading cause of cancer mortality globally. Approximately 340 people die from lung cancer each day, with a 5-year relative survival rate of 25% (3, 4). In China, lung cancer ranks highest in both incidence and mortality among all malignant tumors (5). The incidence and mortality rates of lung cancer continue to rise worldwide. Lung cancer is categorized into small cell lung cancer (SCLC) and non-small cell lung cancer (NSCLC) on the basis of histological characteristics, with NSCLC accounting for 80% to 85% of cases (6, 7).

Precision treatment technologies for lung cancer are advancing rapidly, with immune checkpoint inhibitors (ICIs) emerging as a focal point of research and significantly improving survival outcomes for some lung cancer patients. Particularly for patients with advanced NSCLC who lack driver gene mutations, ICIs used alone or in combination with other therapies have become a first-line standard treatment (8–10). Cancer immunotherapy combats cancer by enhancing the body’s immune system. In recent years, immunotherapy has rapidly developed and is now used alongside surgery, radiation therapy, chemotherapy, targeted therapy, and endocrine therapy to fight cancer. Immunotherapy employs two main strategies: the first is to stimulate the immune system through vaccine therapy to recognize and attack cancer cells (active immunotherapy), and the second is to use checkpoint inhibitors to increase the inhibitory state of the immune system, thereby restoring its ability to eliminate cancer cells (passive immunotherapy) (11). Monoclonal antibodies targeting immune checkpoints, including programmed cell death-1 (PD-1) and its ligand PD-L1, have shown response rates of up to 40% in monotherapy (12).

Given the complex fluid dynamics of tumors and the immunogenic variations within the tumor microenvironment (TME), relying solely on a single biomarker to accurately predict the efficacy of ICIs remains an unstable approach (13, 14). Numerous studies have established a prognostic evaluation framework for NSCLC patients treated with ICIs, integrating multidimensional information from clinical practice, pathological histology, genomics, and radiomics to provide a more comprehensive understanding of treatment prospects (15, 16). Therefore, this study aimed to develop a comprehensive overall survival risk prediction model for patients with NSCLC receiving immunotherapy that incorporates demographic information, clinical characteristics, and laboratory indicators.

## Materials and methods

### Data sources and patient screening

In a prospective cohort study, data were collected from 1478 NSCLC patients recorded in the Chongqing University Cancer Hospital tumor database between January 1, 2019, and December 31, 2022. The inclusion criteria were as follows: (1) aged ≥18 years; (2) histopathologically confirmed NSCLC; (3) receiving at least two cycles of ICI treatment; and (4) complete clinical data. The exclusion criteria were as follows: (1) death within 48 hours of admission; (2) poor treatment compliance; (3) severe underlying diseases (e.g., severe cardiovascular disease, liver or renal failure, or stroke with severe sequelae); and (4) incomplete follow-up records. This study was conducted following the principles of the Declaration of Helsinki and was approved by the Ethics Committee of Chongqing University Cancer Hospital. Written informed consent was obtained from all participants. The flowchart of this study is shown below.

### Clinical factor

In this study, we collected data on demographic characteristics, including age, sex (female and male), body mass index (BMI), and marital status (married and others). Clinical characteristics, including the Karnofsky Performance Status (KPS) score; hypertension, diabetes, and chronic obstructive pulmonary disease (COPD); and clinical information regarding surgery, radiotherapy, chemotherapy, and targeted therapy, were also recorded. Additionally, we collected laboratory variables from patients before and during treatment (every 6 weeks), including hemoglobin (Hb), white blood cell count (WBC), lactate dehydrogenase (LDH), C-reactive protein (CRP), T lymphocytes (T cells), B lymphocytes (B cells), natural killer (NK) cells, the albumin/globulin ratio (ALB/GLB), β2-microglobulin, the CD4/CD8 ratio, the platelet−lymphocyte ratio (PLR), the neutrophil−lymphocyte ratio (NLR), and the lymphocyte−monocyte ratio (LMR).

### Outcomes and follow-up

The primary outcomes were the probabilities of 1-year, 2-year, and 3-year OS. OS was defined as the time from the initial diagnosis to death, loss to follow-up, or the last follow-up. All the subjects were followed up every 6 weeks until the endpoint of follow-up. The last follow-up date was May 31, 2024. The median survival time of patients in this study, along with the 95% confidence interval, was 25.13 (24.9, 25.37) months. We used a combination of active (data collection via phone calls) and passive (information matched through the hospital information system) follow-up methods to obtain and evaluate patient survival outcomes comprehensively.

### Construction of the nomogram

The patient cohort was randomly divided into two groups at a 7:3 ratio: the training cohort with 925 subjects and the validation cohort with 396 subjects. Data from the training cohort were used to develop the nomogram model. To assess the role of each covariate as a prognostic factor for OS, an initial Cox stepwise regression analysis was performed to identify the optimal model. These variables were then included in a multivariate Cox regression model to analyse their independent associations with OS further, thereby identifying key independent risk factors influencing OS. Nomogram construction was based on the stepwise process’s Cox proportional hazards regression model.

### Model performance and validation

The predictive accuracy of the model was measured via the concordance index (C-index), while the area under the receiver operating characteristic curve (AUC) was used to evaluate the predictive performance of the nomogram comprehensively. Additionally, calibration curves were employed for visual assessment, illustrating the consistency between the model’s predicted results and actual observations. Decision curve analysis (DCA) was utilized to further quantify the model’s clinical utility. This method compares the clinical “net benefit” of using the nomogram to guide decisions against two extreme strategies, “treat all” and “treat none,” thereby providing a comprehensive evaluation of the model’s practical application value.

### Statistical analysis

Data analysis was performed via R version 4.3.1. The development and evaluation of the model were conducted with the R packages “survival” (version 3.5–7), “foreign” (version 0.8–84), “rms” (version 6.7–0), “timeROC” (version 0.4), and “ggDCA” (version 1.1). Additionally, a web server for the NSCLC nomogram was developed via the R packages “rsconnect” (version 1.0.2) and “DynNom” (version 5.0.2) (access link):. Missing data were handled via the multiple imputation method from the “mice” package (version 3.16.0). Categorical variables in the baseline data are expressed as frequencies and percentages [N(%)], and continuous variables are expressed as the means (SDs) or medians (IQRs). Differences in demographic and clinical characteristics between the training and validation cohorts were compared via Pearson’s chi-square test for categorical variables and one-way ANOVA for continuous variables. Significant feature variables were identified via stepwise Cox regression and multivariate Cox regression models. All the statistical tests were two-tailed, with the significance level at P < 0.05.

## Results

### Baseline characteristics of immunotherapy patients

A total of 1,321 patients with complete information were included in the study and randomly divided into training (n = 925) and validation (n = 396) cohorts at a 7:3 ratio. The median survival time and 95% confidence interval for the entire cohort were 25.13 (24.9, 25.37) months, those for the training cohort were 25.10 (24.8, 25.33) months, and those for the validation cohort were 25.47 (24.5, 29.43) months. During the follow-up period, 838 patients (63.44%) survived, whereas 483 patients (36.56%) died. There were 1,040 males (78.73%) and 281 females (21.27%), with a mean age of 60.81 ± 9.33 years. Among the patients, 91.29% were married. The majority of patients chose chemotherapy (90.23%). Targeted therapy was chosen by 41.48% of the patients. The proportions of patients who chose surgery and radiotherapy were similar, at 34.75% and 37.62%, respectively. The demographics, clinical characteristics, and laboratory indicators of the total cohort, training cohort, and validation cohort are shown in **Table 1**, and there were no statistically significant differences between the training and validation cohorts.

**Table 1:** Patient demographics and clinical characteristics.

Characteristics	All patients (n=1321)	Training cohort (n=925)	Validation cohort (n=396)	P
Age (years)	60.81 ± 9.33	60.83 ± 9.33	60.75 ± 9.33	0.882
Gender (%)				
Female	281 (21.27)	196 (21.19)	85 (21.46)	0.969
Male	1040 (78.73)	729 (78.81)	311 (78.54)	
KPS	81.02 ± 7.34	81.18 ± 7.25	80.63 ± 7.52	0.210
BMI (kg/m2, %)				
<24	716 (54.20)	498 (53.84)	218 (55.05)	0.823
≥24	536 (40.58)	380 (41.08)	156 (39.39)	
<18.5	69 (5.22)	47 (5.08)	22 (5.56)	
Marriage (%)				
Married	1206 (91.29)	840 (90.81)	366 (92.42)	0.397
Others	115 (8.71)	85 (9.19)	30 (7.58)	
Hypertension (%)				
NO	1011 (76.53)	711 (76.86)	300 (75.76)	0.716
YES	310 (23.47)	214 (23.14)	96 (24.24)	
Diabetes (%)				
NO	1124 (85.09)	777 (84.00)	347 (87.63)	0.107
YES	197 (14.91)	148 (16.00)	49 (12.37)	
COPD (%)				
NO	1272 (96.29)	892 (96.43)	380 (95.96)	0.797
YES	49 (3.71)	33 (3.57)	16 (4.04)	
Surgery (%)				
NO	862 (65.25)	597 (64.54)	265 (66.92)	0.442
YES	459 (34.75)	328 (35.46)	131 (33.08)	
Radiotherapy (%)				
NO	824 (62.38)	575 (62.16)	249 (62.88)	0.854
YES	497 (37.62)	350 (37.84)	147 (37.12)	
Chemotherapy(%)				
NO	129 (9.77)	81 (8.76)	48 (12.12)	0.074
YES	1192 (90.23)	844 (91.24)	348 (87.88)	
Targeted (%)				
NO	773 (58.52)	548 (59.24)	225 (56.82)	0.448
YES	548 (41.48)	377 (40.76)	171 (43.18)	
Hb (g/L)	120.93 ± 21.15	120.51 ± 21.67	121.91 ± 19.87	0.269
WBC (10^9^/L)	7.25 ± 3.46	7.30 ± 3.62	7.14 ± 3.06	0.453
LDH (U/L)^a^	210.00 (178.00, 262.00)	211.00 (178.00, 260.00)	209.50 (179.00, 265.25)	0.753
CRP (mg/L)^a^	9.57 (4.43, 44.39)	10.05 (4.44, 44.85)	9.07 (4.43, 43.92)	0.824
T (%)	947.93 ± 435.43	958.66 ± 453.01	922.86 ± 390.70	0.171
B (%)^a^	112.00 (58.00, 195.00)	110.00 (61.00, 196.00)	114.00 (53.00, 190.25)	0.744
NK (%)^a^	211.00 (134.00, 326.00)	220.00 (134.00, 328.00)	197.00 (133.50, 316.50)	0.183
ALB/GLB	1.20 ± 0.31	1.21 ± 0.31	1.18 ± 0.30	0.110
β2.microglobulin (mg/L)	2.99 ± 1.26	2.99 ± 1.23	2.99 ± 1.35	0.98
CD4/CD8	1.77 ± 0.98	1.77 ± 0.95	1.77 ± 1.05	0.946
PLR^a^	172.19 (126.42, 244.16)	167.94 (123.98, 240.00)	185.55 (132.11, 260.66)	0.012
NLR^a^	3.35 (2.27, 5.27)	3.29 (2.24, 5.27)	3.49 (2.36, 5.24)	0.297
LMR^a^	2.25 (1.57, 3.11)	2.35 (1.59, 3.14)	2.10 (1.52, 3.00)	0.038

a Expressed as the median (M) and interquartile range (IQR).

### Independent prognostic factors in the training cohort

In the training cohort (n = 925), independent prognostic factors were analysed via the Cox proportional hazards model, and the modelling results are shown in **Table 2**. In the univariate analysis, the following variables were found to be significant predictors of OS: age; Karnofsky Performance Scale (KPS) score; BMI; diabetes status; chronic obstructive pulmonary disease (COPD); radiotherapy; targeted therapy; Hb, white blood cell (WBC) count; lactate dehydrogenase (LDH), C-reactive protein (CRP), serum albumin (ALB)/globulin (GLB), β2-microglobulin, the NLR, and the LMR (all p < 0.05). In the multivariate analysis, the independent prognostic factors were age (hazard ratio [HR]: 1.03; 95% confidence interval [CI]: 1.02–1.03), KPS (HR: 0.98; CI: 0.97–0.99), BMI (HR: 0.76; CI: 0.60–0.96), diabetes (HR: 1.47; CI: 1.11–1.94), targeted therapy (HR: 0.80; CI: 0.64–1.00), Hb (HR: 0.98; CI: 0.98–0.99), WBC (HR: 1.06; CI: 1.02–1.09), LDH (HR: 1.00; CI: 1.00–1.00), CRP (HR: 1.00; CI: 1.00–1.01), PLR (HR: 1.00; CI: 1.00–1.00), and LMR (HR: 0.91; CI: 0.83–0.99).

**Table 2:** Univariate and multivariate analyses of overall survival in the training cohort.

Characteristic	Univariable	Multivariable
Age (years)	1.03 (1.02-1.04, p<0.001)	1.02 (1.00-1.03, p=0.010)
Gender (%)		
Female		
Male	1.16 (0.89-1.51, p=0.275)	
KPS	0.96 (0.95-0.97, p<0.001)	0.98 (0.97-0.99, p=0.005)
BMI (kg/m2, %)		
<24		
≥24	0.60 (0.48-0.76, p<0.001)	0.76 (0.60-0.96, p=0.022)
<18.5	1.51 (0.95-2.39, p=0.080)	1.10 (0.69-1.75, p=0.690)
Marriage (%)		
Married		
Others	0.97 (0.64-1.48, p=0.900)	
Hypertension (%)		
NO		
YES	1.03 (0.80-1.31, p=0.835)	
Diabetes (%)		
NO		
YES	1.48 (1.13-1.92, p=0.004)	1.47 (1.11-1.94, p=0.006)
COPD (%)		
NO		
YES	1.82 (1.15-2.90, p=0.011)	
Surgery (%)		
NO		
YES	0.86 (0.69-1.08, p=0.193)	
Radiotherapy (%)		
NO		
YES	0.75 (0.60-0.93, p=0.010)	
Chemotherapy(%)		
NO		
YES	0.87 (0.61-1.26, p=0.470)	
Targeted (%)		
NO		
YES	0.71 (0.57-0.88, p=0.002)	0.80 (0.64-1.00, p=0.049)
Hb (g/L)	0.98 (0.98-0.98, p<0.001)	0.98 (0.98-0.99, p<0.001)
WBC (10^9^/L)	1.09 (1.06-1.12, p<0.001)	1.06 (1.02-1.09, p=0.001)
LDH (U/L)	1.00 (1.00-1.00, p<0.001)	1.01 (1.00-1.01, p<0.001)
CRP (mg/L)	1.01 (1.00-1.01, p<0.001)	1.01 (1.01-1.02, p=0.001)
T (%)	1.00 (1.00-1.00, p=0.489)	
B (%)	1.00 (1.00-1.00, p=0.592)	
NK (%)	1.00 (1.00-1.00, p=0.159)	
ALB/GLB	0.39 (0.27-0.56, p<0.001)	
β2.microglobulin (mg/L)	1.11 (1.05-1.18, p<0.001)	
CD4/CD8	1.06 (0.95-1.18, p=0.269)	
PLR	1.00 (1.00-1.00, p=0.121)	0.99 (0.99-0.99, p<0.001)
NLR	1.03 (1.02-1.04, p<0.001)	
LMR	0.82 (0.74-0.90, p<0.001)	0.91 (0.83-0.99, p=0.034)

### Development of the prognostic nomogram model

**Figure 1:**
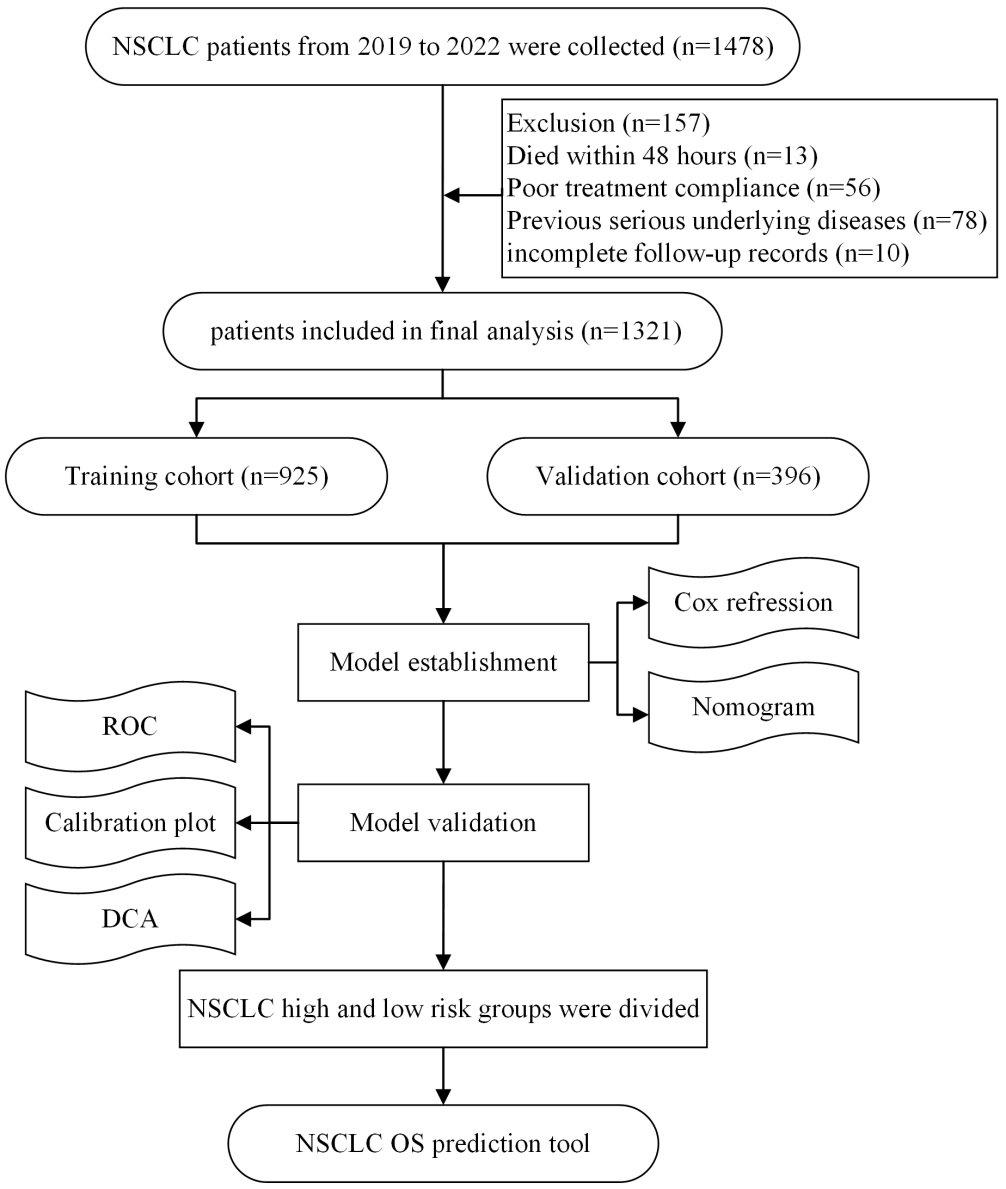
Flow diagram of the study design.

**Figure 2:**
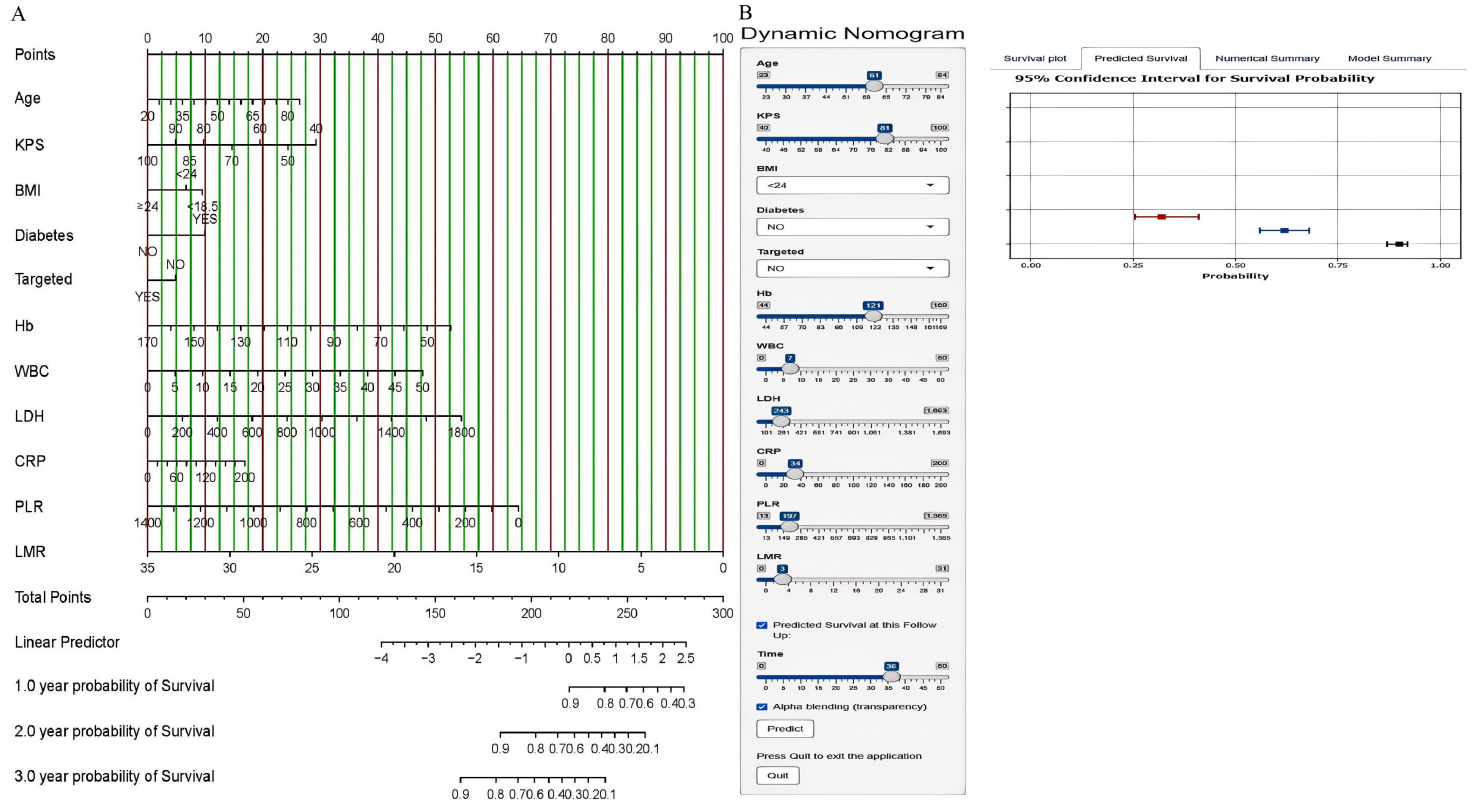
Nomogram for predicting the 1-year, 2-year, and 3-year OS of NSCLC patients in the training cohort **(A)** and the online nomogram tool **(B)**.

To construct predictive models for 1-year, 2-year, and 3-year OS in NSCLC patients, we selected independent prognostic factors identified through multivariate analysis and subsequently developed a nomogram, as shown in **Figure 1**. We converted each selected variable into corresponding scores on the basis of the coefficients estimated from the Cox regression model. These scores were then summed, and the total score was used to estimate the patient’s OS probability from a reference table. We developed an accessible web server for the NSCLC nomogram model (https://icisnsclc.shinyapps.io/DynNomapp/). Users can display the patient’s survival plot and probability by selecting the appropriate indicators and survival time on the left side of the web server interface (**Figure 2**). For example, a patient who is 61 years old with a KPS score of 81, BMI < 24, no diabetes, no targeted therapy, Hb = 121, WBC = 7, LDH = 243, CRP = 34, PLR = 197, and LMR = 3 has 1-year, 2-year, and 3-year survival probabilities of 0.90, 0.62, and 0.32, respectively.

### Model performance and validation of the nomogram

**Figure 3:**
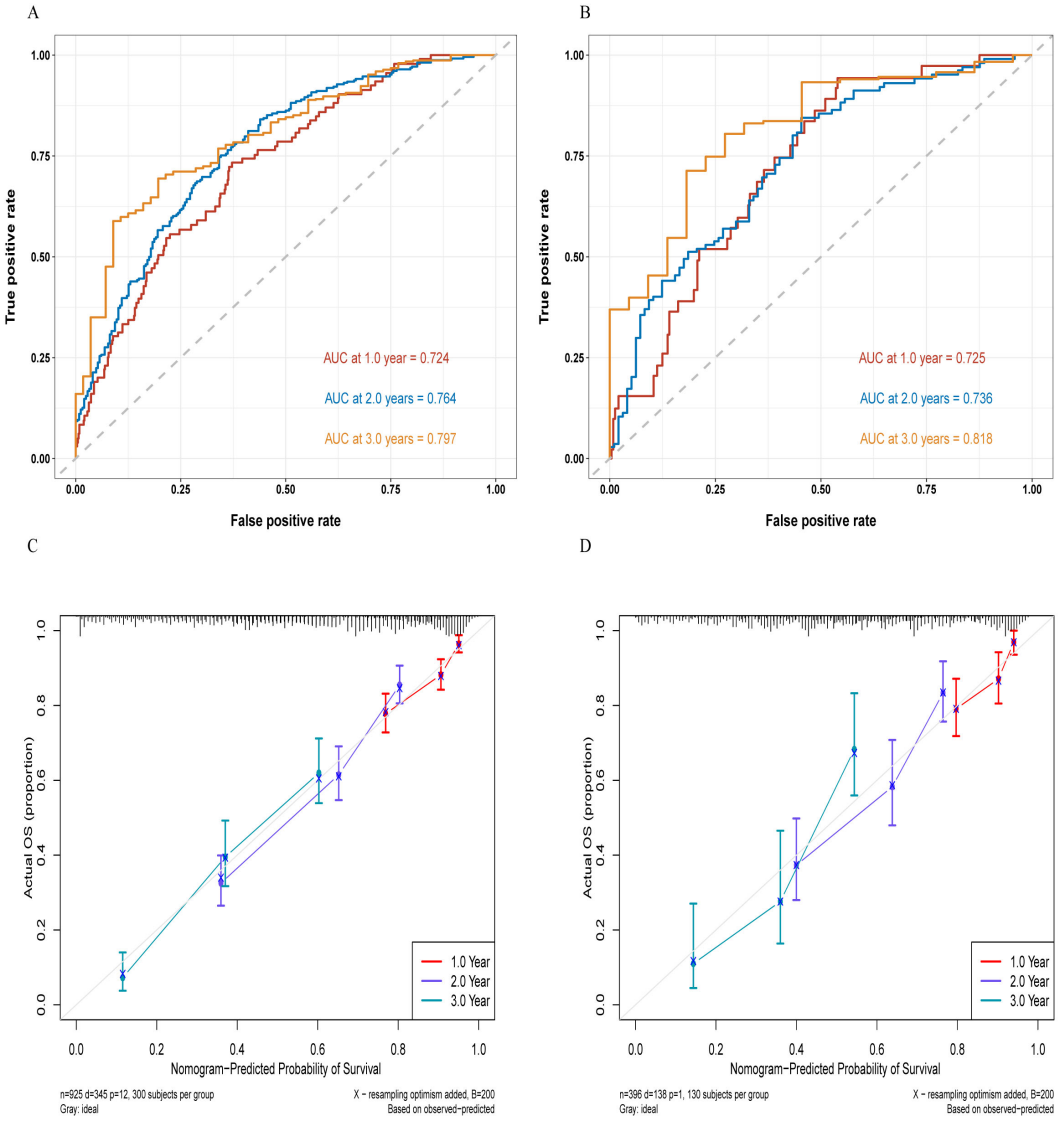
ROC curves for the training cohort **(A)** and the validation cohort **(B)**, as well as the calibration curves for the training cohort **(C)** and the validation cohort **(D)**.

**Figure 4:**
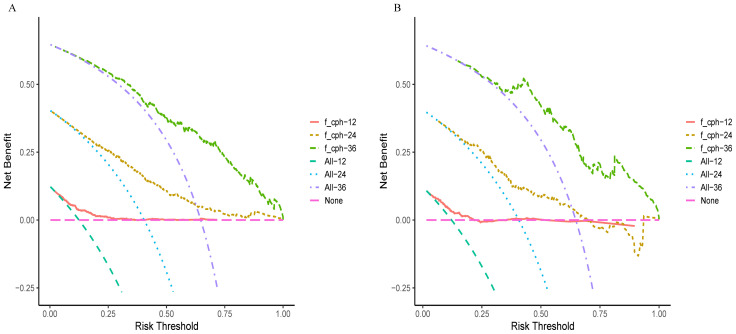
DCA for the training cohort **(A)** and the validation cohort **(B)**.

In the training and validation cohorts, the C-indexes were 0.717 (95% CI, 0.689–0.745) and 0.704 (95% CI, 0.660–0.750), respectively. The area under the curve (AUC) values for predicting 1-year, 2-year, and 3-year OS in the training cohort were 0.724, 0.764, and 0.797, respectively (**Figure 3A**), and those in the validation cohort were 0.725, 0.736, and 0.818, respectively (**Figure 3B**). Moreover, the calibration curves for 1-year, 2-year, and 3-year survival demonstrated good performance, indicating strong agreement between the predicted probabilities and the observed outcomes (**Figures 3C, D**). Additionally, DCA was utilized to evaluate the predictive power of the nomogram. The DCA results for the validation cohort indicated that, except when the predicted probability threshold exceeded 75%, the nomogram provided greater net benefits and more accurate clinical outcome predictions than the prediction that all patients would die or survive (**Figure 4**).

### The risk stratification ability of the nomogram

**Figure 5:**
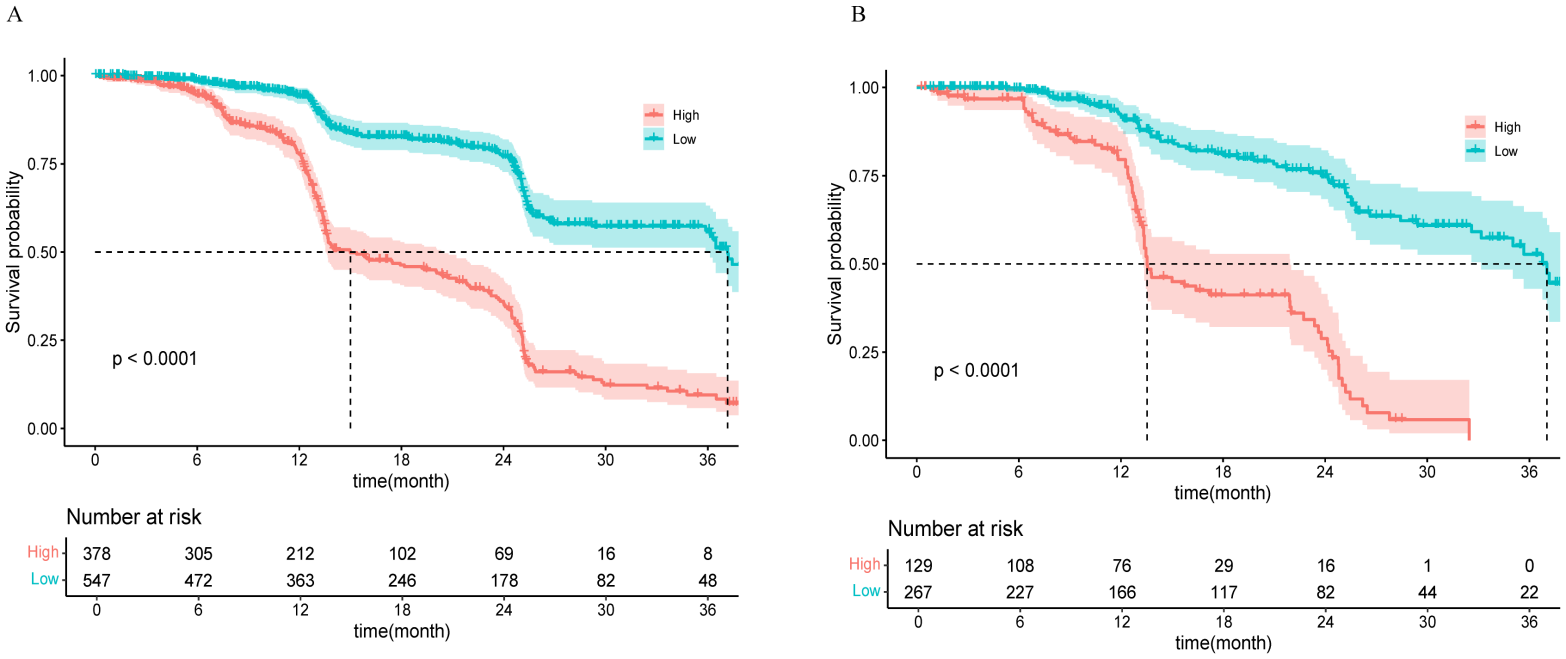
K−M curves for the high-risk and low-risk groups in the training cohort **(A)** and the validation cohort **(B)**.

Using the nomogram model to predict risk scores, a threshold of 0.473 was set via the `surv_cutpoint` function to categorize patients into low-risk (scores below the threshold) and high-risk groups (scores above the threshold). K−M survival analysis of the training and validation cohorts revealed highly significant differences between these two groups (p < 0.0001), as shown in **Figure 5**. In the training cohort, the median survival time for the high-risk group was 15 months (95% CI, 13.53–20.43), whereas that for the low-risk group was 37.17 months (95% CI, 35.87–NA). In the validation cohort, the median survival time for the high-risk group was 13.53 months (95% CI, 13.33–21.93), and for the low-risk group, it was 37.03 months (95% CI, 33.17–NA).

## Discussion

This study aimed to develop an OS risk prediction model for NSCLC patients undergoing immunotherapy incorporating demographic information, clinical characteristics, and laboratory indicators. A nomogram was constructed based on age, KPS, BMI, diabetes status, targeted therapy, Hb, WBC, LDH, CRP, PLR, and LMR. The model demonstrated good predictive ability and was developed as an online NSCLC prediction tool.

Previous studies on predicting OS in NSCLC patients are numerous. Mezquita et al. found that the pre-treatment Lung Immune Prognostic Index (LIPI) was closely associated with poor efficacy of ICIs in patients with NSCLC. LIPI consists of two key indicators: the derived neutrophil-to-lymphocyte ratio (dNLR) and LDH levels, where a dNLR greater than 3 and LDH exceeding the upper limit of normal (ULN) are independent predictors of poor prognosis (17, 18). In patients with advanced NSCLC (aNSCLC), continuing immunotherapy after disease progression was associated with prolonged OS and PFS (19). A prognostic risk model for NSCLC based on seven smoking-related genes demonstrated stable predictive ability, with FCGBP having the highest mutation frequency (20). Additionally, studies have shown that in Asian patients with advanced NSCLC receiving immune checkpoint inhibitors, obesity (BMI) is associated with better OS (18). Cheng Lu et al. reported that CellDiv features (computer-extracted tumor cell diversity features) strongly predict 5-year OS in early-stage NSCLC patients and are related to apoptotic signalling and cell differentiation pathways (21). In 2020, the Mazzaschi team prospectively collected baseline peripheral blood from 109 NSCLC patients receiving continuous ICI treatment to construct the immune effect score (IeffS) based on a tumor−host interaction model. IeffS revealed that elevated PD-L1, reduced CD8+PD-1+ cells, and the absence of NK cells were risk factors for reduced ICI efficacy (P<0.01). In combination with LIPI, it significantly impacted PFS (HR=4.61), OS (HR=4.03), and ICI response rates (P<0.001) (22). A phase III trial in stage I-III NSCLC patients reported a median PFS of 20.8 months for the standard chemotherapy group compared with 31.6 months for the nivolumab group, with 24% of patients achieving pathological complete response (23). However, some previous studies have been limited in their choice of predictive factors, focusing on single dimensions (24–26). In contrast, this study enhances predictive performance and comprehensiveness by incorporating a comprehensive set of predictive factors from three domains.

The nomogram model has been widely adopted tumor prognosis risk assessment, demonstrating excellent predictive accuracy (27). In 2021, Zeng et al. conducted a retrospective analysis of immunotherapy plus chemotherapy data from 130 stage IIIA-IVB NSCLC patients and developed a PFS prediction nomogram based on bone metastasis, the dNLR, smoking status, and PD-L1 expression. The low-risk group had a significantly longer median PFS (P<0.001). The model demonstrated good predictive performance, with C-indexes of 0.725 and 0.688 in the training and validation sets, respectively (28). In 2020, the Hopkins team developed and validated a prognostic tool based on clinical trials to identify advanced NSCLC patients who would benefit from atezolizumab treatment. This tool includes factors such as PD-L1 expression levels, dNLR values, CRP concentrations, LDH activity, albumin (ALB) levels, patient performance status, the period since self-diagnosed metastasis, and the number of metastatic sites. The low-risk group benefited the most, and this study was the first to identify CRP as an OS predictor, with CRP reduction associated with prolonged OS (P<0.001, c=0.66) (29). Other studies have constructed nomogram models based on patients’ general characteristics, histological features, pathological and immunohistochemical results, inflammation, and nutritional indicators to effectively predict OS and PFS in NSCLC patients after surgery (30). Mengmeng Song et al. identified the ALI, LCR, and NLR as the top three inflammation/nutrition indicators for predicting the prognosis of lung cancer patients (31). A study on local tumor response and survival outcomes following combined treatment of stereotactic radiosurgery and immunotherapy in NSCLC with brain metastases revealed that a KPS score of < 80 (p = 0.001) and a lung-specific molecular marker graded prognostic assessment (lung-mol GPA) score of < 1.5 (p = 0.02) were predictive of poorer survival outcomes (32). The variables included in this study are broadly consistent with previous studies, and Hb and WBC count have been newly identified as independent prognostic factors for NSCLC patients. This finding provides a new perspective and an essential basis for evaluating disease prognosis.

The biological complexity and heterogeneity of NSCLC underscore the necessity of personalized treatment. Molecular targeted therapies based on driver gene mutations, such as EGFR, ALK, and ROS1, have achieved significant advances in the treatment of advanced NSCLC. However, various resistance mechanisms, such as secondary mutations and bypass activation, limit the long-term efficacy of targeted therapies. The limitations of single-agent therapies suggest that combination treatments may improve outcomes. ICIs, in combination with chemotherapy, targeted therapy, or radiotherapy, have shown promising results in clinical studies (33). Traditionally, radiotherapy has been thought to kill tumor cells mainly by damaging DNA. However, radiotherapy can also control metastatic lesions outside the irradiated field through the “abscopal effect.” This mechanism involves radiotherapy-induced tumor cell death, releasing tumor-associated antigens, which activate a systemic immune response to control tumor proliferation (34). For patients with unresectable stage III NSCLC and wild-type driver genes, combining radiotherapy and immunotherapy in initial treatment is crucial for significantly improving efficacy (35). The PACIFIC trial was a landmark study that demonstrated durvalumab, an ICI, significantly prolonged PFS and OS in patients after concurrent chemoradiotherapy. The real-world effectiveness and safety of durvalumab are consistent with the results of the PACIFIC trial, further supporting its use as the standard treatment for patients with unresectable stage III NSCLC (36).

The limitations of this study primarily lie in the lack of a multicenter research design and external validation. Although the sample size was relatively large, the findings need to be confirmed in larger-scale prospective cohorts. This limitation may introduce selection bias, thereby restricting the generalizability and representativeness of the results. Future research should aim to establish a multicenter collaborative framework to enhance the comprehensiveness and reliability of the data. Moreover, personalized prediction has become possible with advancements in genomics, translational medicine, and precision medicine technologies. The study found that AI models can accurately predict tumor response and PFS of ICIs in advanced NSCLC by assessing PD-L1 tumor proportion score (TPS). In the future, it may be feasible to offer more efficient and convenient personalized risk assessment strategies by integrating advanced gene testing techniques and the assistance of artificial intelligence (37, 38).

## Conclusion

In this study, we developed an OS risk prediction model for patients undergoing immunotherapy for NSCLC that incorporates demographic information, clinical characteristics, and laboratory indicators. The model demonstrated good predictive ability and was developed as an online NSCLC prediction tool. This tool facilitates convenient and efficient individualized risk assessment, providing real-time prognostic prediction services for healthcare providers and patients.

## Data Availability

The raw data supporting the conclusions of this article will be made available by the authors, without undue reservation.
